# Are Absence Epilepsy and Nocturnal Frontal Lobe Epilepsy System Epilepsies of the Sleep/Wake System?

**DOI:** 10.1155/2015/231676

**Published:** 2015-06-14

**Authors:** Péter Halász

**Affiliations:** National Institute of Clinical Neuroscience, Lotz K. u. 18, Budapest 1026, Hungary

## Abstract

System epilepsy is an emerging concept interpreting major nonlesional epilepsies as epileptic dysfunctions of physiological systems. I extend here the concept of reflex epilepsy to epilepsies linked to input dependent physiological systems. Experimental and clinical reseach data were collected to create a coherent explanation of underlying pathomechanism in AE and NFLE. We propose that AE should be interpreted as epilepsy linked to the corticothalamic burst-firing mode of NREM sleep, released by evoked vigilance level oscillations characterized by reactive slow wave response. In the genetic variation of NFLE the ascending cholinergic arousal system plays an essential role being in strong relationship with a gain mutation of the nicotinic acethylcholin receptors, rendering the arousal system hyperexcitable. I try to provide a more unitary interpretation for the variable seizure manifestation integrating them as different degree of pathological arosuals and alarm reactions. As a supporting hypothesis the similarity between arousal parasomnias and FNLE is shown, underpinned by overlaping pathomechanism and shared familiarity, but without epileptic features. Lastly we propose that both AE and NFLE are system epilepsies of the sleep-wake system representing epileptic disorders of the antagonistic sleep/arousal network. This interpretation may throw new light on the pathomechanism of AE and NFLE.

## 1. Introduction

*The Emerging Concept of System Epilepsies*. After the detronization of the “generalized epilepsy” concept [1] the epilepsy community realized that generalized epilepsies are not really generalized and focal (partial) epilepsies are not as focal as we held earlier. In the same time the emerging view of “epileptic networks” [2] seems to provide a new frame for conceptualising idiopathic epileptic disorders whithout lesional origin or even to those lesional epilepsies which tend to involve extended bilateral cerebral circuits. As Wolf [3] summarized: “Both localisation-related and ‘generalized' idiopathic epilepsies are about to be understood as related variants of system disorders of the brain, with an ictogenesis making pathological use of existing functional anatomic networks.”

Since epilepsy is known to be the a pathological facilitation of normal physiological functions a self-evident idea bystanding long ago in our thinking is that different physiological working systems of the brain provide templates for epileptic disorders endowing them with special features. The idea of the so called “system epilepsies” has been emphasized more and more recently during the discussion of the new epilepsy classification [3–5] and network epilepsies [6–9].

Elevated excitability of a neural system as a transitory developmental feature (usually with genetic origin) or as an enduring propensity to generate seizures during a period of life can be an essential constituent of system epilepsy. Evolution of the concept of system epilepsy seems to be linked especially with the epileptic facilitation of the sensory systems. The new ILAE epilepsy definition [10] requires no more that the epileptic seizure should be “spontaneous” (which would exclude “reflex seizures” or in more general “input dependent seizures”) and defines epilepsy by any “enduring alteration in the brain that increase the likelihood of future seizures.” Reflex seizures fulfill this requirement carrying the trait of epileptic input sensitivity [11].

Epileptic discharges and seizures can be activated by triggering a function the epilepsy is connected with. Classical example is sensory reflex epilepsies (visual stimulation activates epileptically facilitated occipital cortex). In the last years we have learnt more about the elicitability of seizures by several kinds of sensory stimulation and also by mental and physical conditions either in the so called generalized epilepsies [12–14] or in epilepsies caused by dysgenetic cortical malformations [15].

In other words all the stimuli or influences which are specific to elicit a certain function may yield epileptic responses if there is an epileptogenic network in the system. There is now enough evidence to think as common events that sensory or cognitive triggers can yield epileptic manifestations due to facilitation of epileptogenic networks in certain neo- or archicortical structures. The most frequent ones are somatosensory and visual, but acoustic, proprioceptive, and higher level complex sensory triggers targeting certain cortical functions as listening to music, executing certain practices such as reading, playing chess or card games, activating memory traces, or emotional states also exist. All these sensory inputs involve mainly the posterior parts of the brain while the frontal lobes seem to be left out. However, in this paper I try to show that inner influences activating the arousal and the corticothalamic system and consequently the frontal cortical areas also can work as triggers eliciting epileptic manifestations. In general these phenomena support the system or functional network hypothesis in epilepsy.

System epilepsies display a relationship between the physiological function of the system and features of the epilepsy. The system epilepsy concept that can be valid not only for the so-called idiopathic epilepsies but also for epileptogenic lesions may increase the propensity of a system to react with epileptic manifestations as if being “hijacked” by the epileptogenic process [4].

In certain system epilepsies the normal functions of the system may suffer interference from the epileptic manifestations. We have examples for this condition related also to the sleep system. The epileptic encephalopathy characterized by electrical status epilepticus in slow sleep leads to cognitive impairment by the almost continuous discharges during NREM sleep interfering with the plastic functions of sleep [16, 17]. Similarly in Medial Temporal Lobe Epilepsy (MTLE) the hippocampo-limbic spiking during NREM sleep interferes with the memory consolidation through modifying the sharp wave ripple activity during sleep, leading to memory disturbances.

Boosting factors which promote normal to epileptic transformation might be as follows: genetic constellations, developmental time window, and “epileptogenic lesion” in the system.

In this work I will try to put together evidences to discuss how much can we apply the idea of “system epilepsies” explaining two major epilepsies associated deeply with vigilance functions of the sleep/wake system.

## 2. Absence Epilepsy as System Epilepsy of the NREM Sleep Promoting System

Absences are one of the most frequent epileptic manifestations of childhood and young adulthood. Around 3 Hz bilateral spike-wave paroxysms are the most essential features of absences. Absences occur not only in Childhood Absence Epilepsy were it is the only seizure type of the syndrome, but occur in other types of of idiopathic not localisaton related epilepsy syndromes, like Juvenile Absence Epilepsy (JAE), Juvenile Myoclonic epilepsy (JME) and Eyelid Myoclonia with Absences (Jeavons syndrome). Although the pheno- and genotype are different in these epilepsy syndromes the electroclinical symptoms of absences and presumably the pathomechanism are common.

In the following parts the relation of absence epilepsy (and bilateral spike-wave paroxysms) with sleep will be analysed from several aspects: (a) relation of absences with the brain mechanisms promoting sleep and producing sleep EEG characteristics; (b) timing of absences during the wake/sleep continuum; (c) how phasic sensory input influences the occurrence of absence seizures on different level of homeostatic pressure and vigilance level (is phasic shift of vigilance toward NREM sleep promoting absences?); and (d) what is common and different between absences and neurophysiological characteristics of sleep.

### 2.1. Relationship between the Corticothalamic System and Absence Seizures with Bilateral Synchronous Spike-Wave Discharges

Thalamus [20] and later the corticothalamic system [21] were related long ago to the pathomechanism of absences and bilateral synchronous spike-wave pattern. The similarity in the cortical appearance and mechanism of spindling and bilateral spike-wave pattern especially was the main challenge for researchers who wanted to solve the riddle of the bilateral spike-wave pattern. Gloor from the MNI was the pioneer who tried a systematic approach and after a long fruitless debate about the cortical or subcortical origin of the pattern has drawn the attention to the role of the corticothalamic system (named by him as “corticoreticular”) and brought the role of the sleep system into the question [22]. He proposed that spike and wave paroxysms as EEG substrate of absences emerge from the same circuit that normally produces sleep spindles and the former are epileptic transformation of sleep spindles. This idea fertilized the further research until the present days. Kostopoulos [23] showed that the sleep spindles transform to spike-wave discharges when Penicillin (GABA-A antagonist compound) was placed on the cortical surface in experiments with cats. In his later works Kostopoulos [24] provided further arguments on behalf of the spindle transformation theory.

Steriade summarized the research of his own group and others on this topic in his epoch-marking book about sleep and epilepsy [25]. Working with chronically implanted, freely moving, naturally awake, and sleeping cats they showed that in absence like seizures the spike-wave discharges “originate in the neocortex and are disseminated through monooligo and multisynaptic intracortical circuits, before they spread to the thalamus and exhibit generalized features.” The cortical origin have been supported by the Steriade group with several ablational experiments, and they also stressed the focal to widespread propagation dynamics in the seizures that was later confirmed in several human reports and again convincingly strengthened on a rat genetic model by Meeren et al. [26]. This was a complete break with the old “centrencephalic” theory of Jasper and Penfield who hypothesized a midline deep (thalamic) source of the bilateral synchronous spike-wave pattern [27].

Although it is clear now that there are no bilaterally projecting thalamic neurons, the influential centrencephalic theory and the additional “secondary bilateral synchrony” hypothesis of Tükel and Jasper [28] still survived in several clinical publications. Although it is important to stress the cortical origin we have also emphasized the crucial role of the thalamic involvement that borroughs the very special feature of the absence/spike-wave mechanism. According to the studies of Steriade [25, pages 322-348] during cortical seizures with 3 Hz spike-wave complexes RE (reticular) cells show a spike burst and at the same time TC (thalamocortical) neurons inhibitory postsynaptic potentials can be detected. So GABAergic RE neurons participate actively during cortically generated spike-wave seizures by the same circuit as in spindles. Concerning the “wave” component of the spike-wave discharges Steriade was inclined to interpret it not as a GABAergic inhibition but as “disfacilitation,” similar to what we see during the hyperpolarisation phase of down states within the <1 Hz slow oscillation [25, page 357].

Beenhakker and Huguenard [4] in their interesting paper dedicated to mechanisms by which epilepsy “hijacks” certain sleep related circuits. They discuss the relationship between sleep spindles and bilateral synchronous spike-wave paroxysms, reinvestigating this question by contemporary neurophysiological approaches [29, 30]. One of the prevailing views is that GABA-A receptor blockade promotes the switch between spindling and spike-wave discharge [31, 32] by eliminating intra-RE inhibition working in normal sleep spindling.

Nowadays a lot of gene mutations have been shown to play a possible role in the genesis of absences, but we still do not know which are the relevant epileptogenic modifications in the human absence epilepsy. Whatever the concrete mechanisms may be there is strong support to the thesis that normal thalamic functional circuits, working during NREM sleep, offer a template that epileptic circuits use to generate seizures, or saying it another way, normal sleep circuitry is hijacked to generate epilepsy.

For example, RE neurons contain GABA-A receptor beta 3 subunit, while those expressed by TC neurons do not. Genetically removing the beta 3 subunit from mice eliminates intra-RE inhibition but does not affect RE-to-TC inhibition. Beta 3 knockout mice have thalamic oscillation which are more robust and synchronous (like in epilepsy) and these mice have an Angelman syndrome like phenotype including absence like seizures. Thus reducing intra-RE inhibition is likely to contribute to absence like seizures. Electrical coupling among RE neurons also likely contributes to hypersynchrony among RE neurons during spike-wave discharges [33, 34]. Congruently carbenoxolone inhibitor of gap junctional communication reduces the number and duration of spike-wave discharges in rat and mouse models of absence epilepsy [35].

Many factors can influence RE neurons excitability, but the most likely factor playing role in promoting absences with spike-wave pattern is probably the excitatory input originating from the cortex that may transform spindle oscillation into 3 Hz oscillation. Ample variations are possible via the up- and downregulation of excitatory/inhibitory balance in cortical, thalamic, and reticular neurons. The corticothalamic circuit also may be influenced via several ascending and descending pathways from the brain stem and the cortex. Therefore, it is not surprising that the same distortion of functions resulting in epileptic spike-wave discharges in the system could stem from influences from different key points in the network.

### 2.2. How Vigilance Level Changes across Sleep/Wake Cyle and the Circadian Cycle Influences the Release of Absences?

A very close relationship between the level of vigilance and expression of spike-wave discharges has been shown from the seventies of the past decade. Early studies clearly proved that spontaneous paroxysms are promoted by transitory decreases of vigilance after awakening in the morning, after lunch, in evening sleepiness, during boring situations [36–38], and in experimental depression of reticular arousal functions [39]. Whole night studies have shown the same: 3 Hz generalized spike-wave discharges (SWD) occur selectively in the transitional periods between NREM, wakefulness, and REM sleep [40–42]. The same tendencies have been shown also in animal studies [43, 44]. The spike-wave pattern is absent in REM sleep in both humans and animals [25, pages 334-335]. However, in those periods where REM sleep is not fully developed and elements of NREM sleep (sleep spindles and/or vertex sharp waves) or wakefulness (alpha spindles) are mixed, SWD paroxysms may appear [40].

There are very few works aiming to study how absences are distributed, under the influence of circadian rhythms. This approach requires a highly sophisticated experimental setup, because absence epilepsy is very closely associated with vigilance level changes. Therefore, all the studies looking for clock-time circadian seizure occurrence of absences without controlling the actual vigilance state have serious limitations confounding the possible circadian effect with the vigilance effect. Tomka [47] registered 30 patients with absence epilepsy by 37 telemetric and all night polygraphic recordings. The preponderance for absences was the highest in “hypersynchronous alpha state” contrasting with full wakefulness. Smyk et al. [48] investigated in the Wag/Rij rat model of absence epilepsy the spontaneous rhythm of seizure occurrence; they registered the motor activity, the circadian parameters, the spike-wave events, and sleep-wake states. Spike-wave discharges were preceded mostly by passive wakefulness and slow wave sleep. Results suggested that the mechanism of spike-wave generation is independent from the circadian timing system. Zarowski et al. [49] studied the circadian distribution and sleep/wake patterns of children's generalized seizures. Absence seizures occurred predominantly in wakefulness, 9 am to noon and 6 pm to midnight, but the preceding state of vigilance was not scrutinized.

Pavlova et al. [50] measured in five patients with generalized epilepsy (4 with juvenile myoclonic type) the occurrence time of interictal spike-wave discharges parallel with their hourly plasma melatonin level to evaluate circadian rhythmicity. Most spike-wave discharges occurred during NREM sleep (NREM/wake ratio: 14 : 1). In two patients who had NREM in all circadian phases there was an apparent circadian variation in spike-wave discharges but with different phases relative to the melatonin peaks. Thus this pilot study supports the existence of a circadian modulation of discharge occurrence beyond the obvious NREM dominance in the distribution; however, it should be emphasized that only the interictal discharges, not the absence type 3 Hz spike-wave paroxysms, were measured.

**Figure 1:**
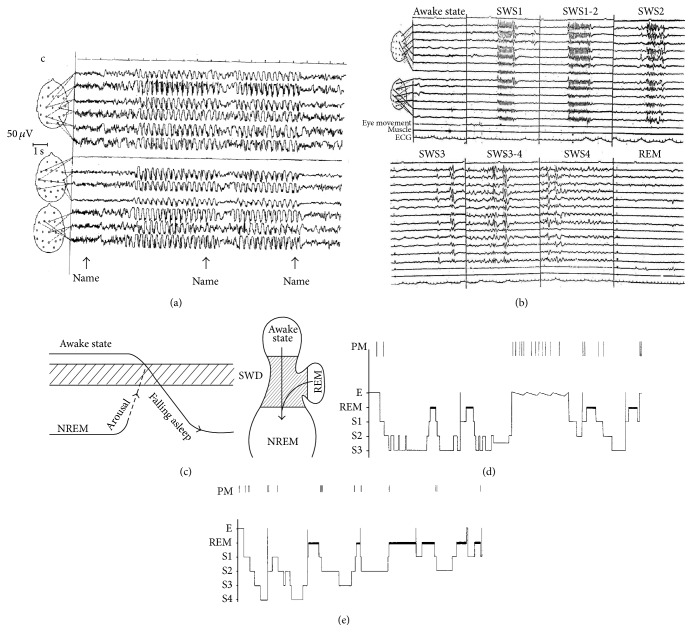
Distribution of generalized spike-wave discharges in different vigilance levels. (a) and (b) In absence epilepsy elevating the vigilance level from NREM sleep by calling the name of the patient first results in transitory activation of the discharges, and later the application of the same stimulus inhibits the discharges. As seen in (c) insert shows the optimal decrease of vigilance level which activates the discharges, while increased arousal and deepening of sleep equally inhibit them. The critical vigilance territory is situated between sleep and wakefulness and NREM and REM, as shown in the left insert. The highest activation have been experienced in shifts from wakefulness to NREM sleep and from REM to NREM sleep (arrows). (b) Shows the typical patterns of spike-wave activation during night sleep. During full wakefulness and REM the spike-wave discharges are absent. During the course of falling asleep (stage 1, superficial stage 2) spike-wave discharges show the ictal absence type activation (even if in awake state they were not present). In deeper sleep (stages 3-4) the typical 3 Hz spike-wave pattern became disrupted; later we see more polyspike-wave forms. (d) and (e) are typical spike-wave activation conditions in absence epilepsy in two patients: in falling asleep, in transitions from wake to NREM or from REM to NREM, around transitory awake state, and before and after awakening in the morning.

By clinical observations absences were considered to be essentially awake events; however, several studies have shown that absences are closely related to decrease of the vigilance level even within the span of the awake domain and further decrease of vigilance to stage 1 until stage 2 [51, 52]. There is evidence from several studies that absences can be stopped by sudden elevation of vigilance by arousing stimulation [53]. The vigilance level dependency of absences explains the distribution of them throughout the 24-hour sleep/wake cycle. Important part of absences may occur hidden in the night during light NREM sleep or around falling asleep and awakening from sleep or associated with the shifts around REM/NREM changes (allways related to the “critical vigilance level” described earlier). The critical preferential zones for absences are characterized not purely by decrease of vigilance level but also by an increased rate of oscillations of the vigilance level to and fro, from higher to deeper level of vigilance and vice versa [54] (Figure 1).

### 2.3. Can Absences Elicited by Experimental Manipulation of the Hypothalamic Sleep Promoting System?

Further support that absence is epilepsy of the sleep system is given by the study of Suntsova et al. [55] working with Wag/Rij rats who have elevated genetic risk for absence epilepsy. They have shown that absence-like seizures could be elicited in the nonepileptic animals by fractional activation (electrical stimulation) of the sleep promoting hypothalamic neurons.

### 2.4. Are Spike-Wave Discharges Elicitable by Sensory Stimuli and Related to the Type of Arousal Driven Phasic Events?

Looking into the dynamic features of these peculiar transitional states of vigilance, they are characterized by high amount of oscillations consisting of bidirectional fluctuations either toward sleep or toward arousal. Applying sensory stimulation to manipulate and control the vigilance level oscillation we have shown that the majority of the “spontaneous” spike-wave paroxysms occur during fluctuations toward NREM sleep compared to fluctuations toward wake of REM state [54]. Application of sensory stimuli increased the fluctuations parallel with increasing the number of spike-wave paroxysms.

Later, after the recognition of CAP, sleep EEG analysis of 10 primary generalized epileptic patients, Terzano et al. [56], showed prevalence of SWD during CAP. Sleep EEG analysis of 10 JME patients [57] revealed that spiking rate was significantly higher in CAP A phase compared to non-CAP and showed strong inhibition in CAP B phase. These data strongly confirm previous results supporting an association of the sleep level fluctuations and the appearance of SWD and also supports the role of shifts toward slow wave sleep.

**Figure 2:**
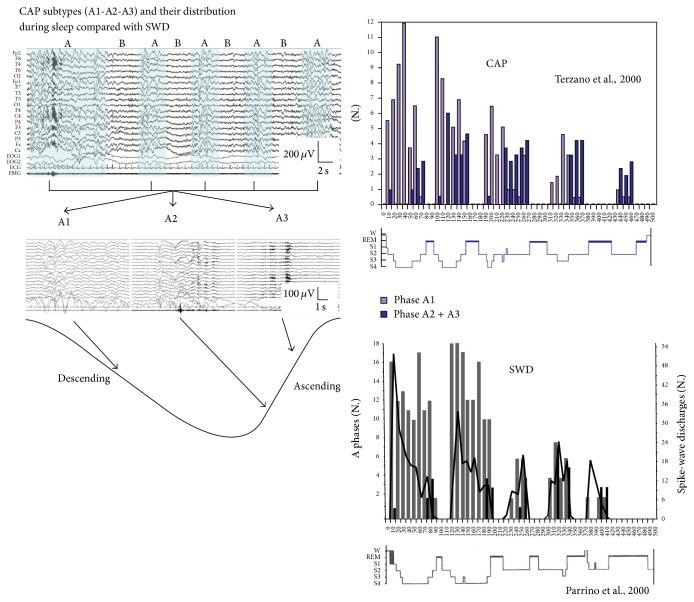
Distribution of CAP subtypes across a sleep cycle and the typical distribution of the subtypes from cycle to cycle, compared with the distribution of spike-wave discharges (after Terzano et al. [18] and Parrino et al. [19]).

In idiopathic generalized epileptic patients, 70% of SWD were in A1, 24% in A2, and 6% in A3 [19]. Consequently, the amount of SWD was the highest in the first cycles, declining later, simultaneously with the decay of delta power from evening to morning (Figure 2). The CAP-related activation of SWD was 3 times higher in descending slopes, dominated by subtypes A1, in contrast to ascending slopes containing higher amounts of A2 and A3 events.

Koutroumanidis et al. [58] studying frontal and generalized spike-wave discharges in 13 children with absence epilepsy have found that generalized discharges tend to appear during periods of sleep fluctuations, especially when sleep shifted from B to A phase of CAP. This finding is again very much congruent with the earlier experience: spike-wave discharges are gated by the fine graded shifts toward NREM sleep.

These studies have shown a close link between spike-wave paroxysms and those phasic events which are sleep promoting (A1 reactive phases of CAP). The interrelationship between spike-wave paroxysms and NREM sleep induction is congruent with the neurophysiological data about the underlying dynamical changes in the corticothalamic system during spike-wave discharges. Increasing evidence points to the role of frontal lobe in NREM sleep slow oscillations including CAP A phases [59] and the same is true for spike-wave discharges. Therefore, the induced spontaneous shifts toward NREM sleep promote the manifestations of absences. The well-known activating effect of sleep deprivation and sleep per se is probably related to the same mechanism [60].

Therefore, the frequently emphasized association of absences with arousal [61] should be treated with caution, since even when the absences were elicited by arousal influences, a more fine graded analysis of the situation shows that absences are linked not to the arousal, but closely correlated with the reactive sleep like antiarousal annswers [62]. The strong correlation between absences and reactive (evoked) sleep slow waves [51] seems to be a strong argument supporting the connection between absences and shifts toward NREM sleep.

### 2.5. What Is Common and Different between Neurophysiological and Neuroimaging Characteristics of Absences and Sleep

For the hypothesis that absence is epilepsy related to the NREM sleep system it is important to discuss what is common and what is different between sleep and absences and what is characteristic for “loss of consciousness” in sleep and in absences.

Studies on the impairment of consciousness in absences [63, 64] revealed that it is far not global and uniform. The impairment is from patient to patient different, fragmentary and involves only “pieces” of consciousness. The individually variable cortical pattern of activation/deactivation in terms of the positive and negative BOLD (Blood-Oxygen-Level-Dependent) signals congruently supports this statement [65–67]. The most frequently affected pieces are perception, cognition, and motor performance.

The variable and fragmentary nature of deficits in cortical functions and consciousness can be interpreted as being congruent with the also variable local differences in slow wave activity, sleep spindles [68], and gamma bursts [69] during NREM sleep. If we consider that epileptic transformation of the suppression-burst activity is initiated not synchronously and with not equal intensity in the whole corticothalamic system especially at the beginning of the paroxysms, it is very similar to the differences in time and space of the development of the sleep process under physiological condition; however, this process is going on much faster during absences.

The impairment of consciousness during sleep and absences is not identical. First of all the obvious motor output inhibition is conspicuous during absences while in sleep, at least in NREM sleep motor output is not under inhibition. The ongoing mental activity which can be revealed if we arouse the sleeper is never present during absences, but after the episode it is regained immediately. The difference is even more complex if we compare the electrical cortical activity in sleep and absences. In NREM sleep we have more or less synchronized frontally dominant slow waves and central spindling activity, while during absences synchronous spike-wave discharges cover the whole scalp with definitive frontal dominance. The underlying events during the “spike” component proved to be unequivocally a pronounced glutamatergic burst discharge both in cortical and in thalamic relay cells. The “wave” component was previously viewed as summated inhibitory postsynaptic potentials attributed to GABAergic inhibitory process in pyramidal cortical neurons. Later Steriade et al. [70] raised that instead of active inhibition a “disfacilitation” is present during the “wave” component.

Comparing with NREM sleep where the brain connectivity is decreased [71], during absences the brain functional connectivity increases; however, the connectivity is less complex [72].

PET studies show that metabolic activity and blood flow are globally reduced in NREM sleep compared to resting wakefulness [73–76] most likely due the hyperpolarized phase of the slow oscillation, during which synaptic activity is essentially abolished. Activation is reduced in all types of association cortex and relatively preserved in primary sensory cortices. NREM sleep is a relatively dynamic state compared with absences, with several local aspects and phasic events related to slow waves, spindling, vertex waves, K-complexes, having their own dynamism being more and more explored, by EEG-fMRI, and source localisation methods [77–79], showing dramatic reorganization of brain connectivity architecture between NREM sleep and wakefulness.

The Discovery of the Resting State or Default Mode Network (DMN). Raichle et al. [80] described, first by PET studies, a peculiar network which appeared when the goal directed thinking was suspended and disappeared when the brain started to be engaged by different goal directed, and activities, triggering other specific networks. The DMN proved to be associated with inner thoughts serving self-processing, day dreaming, free associations, and preserving a free floating awareness of our environment. Besides the stereotype interindividual appearance of the DMN network, it has been shown that parallel with the switch between BOLD changes of DMN and goal directed networks, gamma band activity is also switching between them in a spatially specific way [81].

The work of Sämann et al. [82] showed that resting state network can be followed from wakefulness to slow wave sleep; however, with increasing sleep depth, contributions of the posterior cingulate cortex, retrosplenial cortex parahippocampal gyru,s and medial prefrontal cortex to the resting state network decrease.

Similar but less pronounced changes were shown in the posterior cingulate and retrosplenial cortex to increased sleep pressure during wakefulness [83].

During absences Doppler ultrasonography of the middle cerebral artery has demonstrated a reduction in blood flow during absences [84]. Single Photon Emission Computerized Tomography (SPECT) studies in experimental animals and in humans showed cortical decrease and thalamic increase of blood flow during absences [85]. FDG-PET studies have shown thalamic activation and cortical deactivation during absences [86]. On functional Magnetic Resonance Imaging (fMRI) studies thalamic structures have shown positive BOLD activation while over wide cortical fields patchy mixed positive and negative BOLD activation has been observed, with negative preponderance [87]. Thus functional neuroimaging studies provide us with a congruent evidence for multiple local functional deficit zones in the cortex during absences, but we do not know whether the fragmentary nature of deficits in conscious awareness during absences can be related somehow to these neuroimaging findings.

The first fMRI studies have shown consistent thalamic BOLD positive activation, but cortical changes at that time were not clear. Archer et al. [88] observed a deactivation in the posterior cingulate gyrus and bilateral parietal and anterior frontal regions. Aghakhani et al. [87] found a response in the thalamus in 80% of patients but described an inconsistent pattern of activation and deactivation in frontal and posterior areas. Later Gotman et al. [89] made a group analysis from the same material. The activations were most significant again in the thalamus bilaterally and also present in the mesial frontal region, in bilateral insulae and in the cerebellum. The deactivations were surprisingly consistent with the findings of Archer et al. [88]. The pattern of deactivation, including posterior cingulate and bilateral parietal and anterior frontal regions, was strikingly similar to the regions involved by the default mode network. Later Moeller et al. [66] studied the activation during the time course of absences dynamically and compared across several absences of the same patient. This study showed that BOLD spatial and temporal response patterns were very consistent between discharges of the same patient but quite different from patient to patient. They have shown a deactivation of the DMN network consistently some seconds before the start of the absence which lasted and an initial circumscript frontal positive BOLD activation at the beginning or shortly after the absence. The default mode network inactivation exceeded the duration of the absences.

Concerning the initial appearance of the resting state network before absences [66] we can raise the possibility that it represents the precipitating vigilance level decrease found with other methods before the absences. Similar speculation is given by Carney and Jackson [90] who speak about the first appearance of default mode network (DMN) before the EEG discharges start, as a “permissive cortical event” known to be required for the occurrence of the clinical and/or EEG paroxysm, which could be identical with the precipitatory decrease of vigilance emphasized in the previous chapters.

In NREM sleep the recurrent cortical disfacilitation during down states together with high-level cortical activity during up states during slow oscillation has serious consequences for sensory transmission (Steriade [45]). The main one is that the thalamocortical sensory processing during deep slow wave sleep is not steady [91]. This could be shown by experiments where the averaging of sensory evoked potentials was grouped according to the up and down states of <1 Hz slow wave oscillation in deep sleep of animals. By this method, the amplitudes of cortical somatosensory evoked responses were found to be different: high, although not reaching wakefulness-like levels, during the up states (confirming the possibility of detecting meaningful events even in deep sleep) and low during the disfacilitation of the down state. This lack of stationarity may impair sensory integration during deep slow wave sleep.

Another observation reveals continuous alternation of activation and deactivation of cortical functions in time (not in space) approximately in every 1000–1500 milliseconds. It is the alternation depolarized (up state) and hyperpolarized (down state) phase of <1 Hz slow wave activity during deep sleep.

We do not know the functional meaning of the recurring alternation between the wake level activation during up states and complete loss of activity during down states in deep sleep. Destexhe et al. [92] raise the question “are corticothalamic up states fragments of wakefulness?” and argue supporting this possibility. They demonstrate the similarity between the persistent depolarization of wakefulness and the up states during the >1 Hz slow oscillation upstate of the cortical and thalamic relay neurons. They think that the shortness of the depolarization do not allow the gain of conscious awareness that probably requires an uninterrupted stream of activation. At the same time they show that the T-type Ca^2+^ channel-dependent bursts of action potentials that initiate each up state in corticothalamic neurons might act as triggers for synaptic and cellular plasticity in corticothalamic networks. This is consistent with behavioral experiments suggesting that slow oscillation up states provide a context for the replay and possible consolidation of previous experience.

In the absence the loss of contact with the outer world may have a several additional reasons, because the bursting mode interrupts the continuous flow of information from the environment to the cortex and the inhibition or disfacilitation during the “wave” component involving the cortical association areas decreases the possibilities of cortical elaboration. On the other hand if we consider resting state network as an omnipotent floating awareness to our environment, without being involved in any specific task, the invading spike-wave discharges understandably destroy self-awareness. However, sensory stimuli may have an awakening effect in sleep and a disruptive effect in absence seizures too.

Gotman and Kostopoulos [93] in and update of neural correlates of loss of consciousness in absences raise the possibility that the “deactivation of the DMN may fully or partially be responsible for diminished self-awareness during spike-wave discharges, together with the presence of an abnormally active thalamus, and this may contribute to a partial blocking of sensory information reaching normally the cortex, thus reducing the awareness of the external world.”

### 2.6. NREM Sleep Burst Firing Mode and Absences Share Common Physiological Background

In the above chapters data and arguments were provided in advance for the thesis that NREM sleep and absences share common physiological background.

(a) Investigations supporting that NREM sleep spindles originating in burst-firing working mode may transform into spike-wave paroxysms under the influence of several input changes of the system under genetic influences or due to neurotransmitter effects were described. The key mechanism by which spindling in NREM sleep switches to spike-wave paroxysms seem to be the mutual inhibition between RE neurons, regulating the level of the output inhibition exerted on the thalamic relay cells. If the output inhibition from the reticular neurons is high (intracellular inhibition low) reticular thalamic relay cell inhibition will be more effective, leading to hypersynchronization in the form of spike-waves.

(b) I summarized those sleep microstructural studies which have shown that phasic inputs shifting sleep towards NREM sleep promotion (expressed by reactive slow waves) may have absence/spike-wave discharge promoting effects, while classical arousals, full awake state, and REM inhibit them. The conclusion is that the sleep induction momentum (phasic shift toward NREM sleep) has an absence promoting effect.

Therefore, the slogan of Steriade: “sleep and epilepsy are bedfellows” is really very witty here.

These data strongly support the hypothesis that absence epilepsy with spike-wave pattern is underpinned by the burst-firing working mode of NREM sleep and can be elicited by phasic activation induced shifts toward NREM sleep at the level of superficial sleep EEG.

## 3. Nocturnal Frontal Lobe Epilepsy (NFLE) as a System Epilepsy of the Arousal System

Nocturnal frontal lobe epilepsy is a recognized epilepsy syndrome. This syndrome is not identical with any other “frontal lobe epilepsy.” Therefore, all considerations in this chapter are not valid for frontal lobe epilepsy in general.

Majority of patients with NFLE have sporadic seizures with unknown origin; a minority of them have mutations of the nicotinic acetylcholine receptor subunits [94]. The electroclinical symptoms of the two groups are identical irrespective of their genetic origin [2]. The exclusion or inclusion of cases with lesional aetiology is an open question under debate. In this paper lesional patients will be excluded.

All the studies analyzing the distribution of NFLE seizures across the sleep/wake cycle emphasize their overwhelming occurrence during NREM sleep and only rare or even exceptional appearance during wake state. All the seizure types in NFLE, regardless of their complexity, are preceded by different degree of EEG/EMG/autonomic and behavioural arousal signs [95].

### 3.1. Seizure Semiology as Symptoms of Arousal-Alarm-Frenetic Panic: A Parallelity in NFLE and AP

The NFLE seizures are usually characterized as “hypermotor” because their most conspicuous feature is a movement storm. However, seizure symptoms appears in several forms in an increasing order of severity from simple, seemingly spontaneous fragments of banal motor night behaviour related to arousal without awakening until motor, autonomic, and emotional expressions of frenetic alarm and panic.

Recently Derry et al. [95] reinvestigated the semiology of video-EEG detected seizures in 63 NFLE patients and compared them with 57 arousal parasomnia events. They identified three fundamental patterns, but 79% of the registered events comprised a composite of more than one pattern. The basic patterns were as follows:

1. “Arousal behavior” which was the first (or only) element in 92% of all events: simple arousal behavior including eye opening, head elevation, staring and face rubbing, yawning, stretching, moaning, and mumbling.
2. “Nonagitated motor behavior” (present in 72%): sitting forward, manipulation of nearby objects, and searching (orienting) behavior. Standing and walking was only occasionally registered (patients were mounted with electrodes). Facial expression was impassive or perplexed and coherent speech fragments were also common.
3. “Distressed emotional behavior” (51%): behavior of fear and anguish indicated by the facial expression and speech content. Patients were sitting or standing up, screaming, and expressing frantic behavior. Attempts of restrain evoked aggressive response.

Comparison of NFLE and AP in the Derry study showed that in 79% of AP the events began with arousal behaviour and in 65% of the events followed by more dramatic manifestations, NFLE seizures were preceded also in 49% by arousal behaviour. In 51% of NFLE patients the start was abrupt (without arousal behaviour) and it was indistinguishable from that seen in 21% of parasomnia cases. Tachycardia was prominent feature in both groups.

The events were in 39% triggered by an external (noise) or internal (cough or snore) stimulus in the parasomnia group whereas in the IFNLE group trigger stimulus was identifiable only in 8%.

In the NFLE group environmental interactions were present only in 11% of seizures and coherent speech was rare, if present it was frenetic and not interactive type.

Parasomnias terminated in 74% with NREM sleep or with wakefulness (26%), while NFLE seizures awakened the patients in 88%.

We can draw several lessons from this study. The symptoms of NFLE seizures and parasomniac events are astonishingly overlapping and follow a parallel severity order. The most frequent common aspect is behavioural and autonomic arousal culminating in the third degree of behavioural pattern what we call in NFLE hypermotor seizure and in arousal parasomnia night terror. Eliciting factors by external or internal disturbances and communication with the environment were most frequent in AP compared to NFLE.

After this study the earlier classification of AP events by Broughton [96] into three forms, “confusional arousals,” “sleepwalking” or “somnambulism,” and “sleep terrors,” seems to be outworn and to speak about a continuum of seizure symptoms, instead of delineating distinct groups of behavioral phenomena, is more established. The similarity of the symptoms continuum NFLE and arousal parasomnias (AP) increases the probability of a common brain mechanism of these disorders, as being an arousal disorder (recognized by Broughton coining the term “arousal parasomnia”), regardless of their epileptic or nonepileptic origin. The meanwhile established genetic links between NFLE and AP [97] also supports a shared brain mechanism linking together these two disorders.

Another aspect of the Derry study is that in the light of the data provided by them the usual characterisation of NFLE seizures as simply “hypermotor” does not catch the essence that the increasing complexity of the seizures from fragments of banal arousal behaviour through nonagitated motor until serious alarm behaviour with emotional expressions may reflect different degree of arousal. This interpretation would integrate the seemingly different kind of manifestations and the consequent participation of autonomic components strongly supports this view.

### 3.2. The Role of Arousal in NFLE Reflected by the Dynamics of Sleep Microstructure

Arousability and reactivity to sensory inputs are essential features of sleep differentiating it from coma, keeping the sleeper in a certain contact with the environment. Thus arousal from sleep has an important biological function serving reversibility of sleep and alarm function to wake the sleeper in the case of danger.

It was recognized that sleep is scattered by different degrees of microarousals. Earlier these arousals were considered to be harmful perturbations, disturbing sleep. After understanding more about sleep microstructure we have strong evidences about the regulating role of arousals in sleep [98, 99].

We consider nowadays arousal without awakening (microarousal) as inherent part of dynamic organisation of sleep in the frame of the Cyclic Alternation Pattern (CAP) system. Subtypes of CAP A phase (A1-A2-A3) represent responses to external or internal input sources reflecting the degree of homeostatic pressure. A3 and partially A2 reflects microshift toward REM or awake state and A1 toward NREM sleep (the term “arousal” is used in the case of A1 phase in an extended meaning. The proper term would be “activation with antiarousal feature”).

A characteristic feature of nocturnal frontal lobe epilepsy is that seizures are related to sleep and linked to microarousal events [100]. However, the precise relationship with CAP A phases according to their subtypes (A1-2-3) is not quite clear. More than three thirds of NFLE attacks occurred from slow wave and 28% from stage 2 sleep in the study of Parrino et al. [101]. In studies of Derry and Montagna the attacks of NFLE occurred during stage 2 of NREM sleep [102, 103].

The frequency of the attacks throughout sleep cycles was the highest in the first cycles and followed the homeostatic decay of slow wave sleep across the night. CAP time, CAP rate, number of CAP cycles, and duration of CAP sequences and especially CAP A rate were all elevated in NFLE patients compared to normal controls [101]. These microstructural features of sleep of NFLE patients point to a continuously higher level of arousal activity during NREM sleep, besides the overt arousal related motor seizure elements [104]; however, it is not clear whether the seizures are promoted by these changes or these changes are the consequences of seizures. The increased CAP rate, the increased frequency of CAP responses, and the sleep instability described in patients with NFLE favour the first possibility.

### 3.3. The Hypersensitivity of the Arousal System Is Underpinned by the Epileptogenic Mutation of the Ach Receptor in ADNFLE

A familiar clustering of NFLE cases has been observed long ago and in 1995 Scheffer et al. [94] described the familial variant of the disorder (autosomal dominant nocturnal frontal lobe epilepsy, ADNFLE) with autosomal dominant inheritance. The underlying cause was mutation of the nicotinic acetylcholine receptor a4 (CHRNA4) and b2 (CHRNB2) subunit on the 20q13 and 15q24 chromosome. Patients exhibiting hitherto explored genetic abnormalities represent the small minority of the cases characterized by the same phenotypical features [95].

Acetylcholine (Ach) plays an important role in cortical activation of the frontal cortex during arousal [105, 106]. Thalamus and cortex are important structures being rich in cholinergic fibers, originating from the nucleus basalis of Meynert providing a most robust cholinergic input. It is important to know that not only the transsynaptic but also the nonsynaptic release of Ach should be taken into consideration [107]. The nicotinic acetylcholine receptors are mutated in ADNFE; therefore, it is assumed that the cholinergic arousal system might be pathological in these patients. The receptors, which are mutated in ADNFLE, are preferentially expressed in the thalamus and mesencephalic tegmentum belongs to the ascending arousal system and in the frontal cortex [108]. A statistically significant increase in nAChR density was observed in the group of ADNFLE patients in a region including the mesencephalon and an adjacent part of the diencephalon (superior area of the epithalamus) and in the cerebellum [46, 109]. The finding of an increased number of nAChRs in the regions of the epithalamus (medial habenular area) and the interpeduncular nucleus (IPN) seem to be particularly interesting in the context of a sleep disorder. These structures are part of the limbic system outflow into the brainstem [46, 109]. They are linked by the fasciculus retroflexus, a cholinergic projection from the medial habenula to the IPN. The IPN projects to the ventral tegmental area and to reticular and tegmental brainstem nuclei, particularly to the laterodorsal tegmental nucleus (LDT), which is part of the ascending cholinergic reticular activating system, involved in arousal regulation IPN have important projections to the mediodorsal thalamic nucleus, which projects to prefrontal cortex. The disruption of the fasciculus retroflexus was shown to alter non-REM sleep duration [110] and also controls the integrity of REM sleep. The IPN could be implicated in the sleep/wake cycle primarily through its connections with the LDT, which during non-REM sleep releases Ach in the thalamus at the time of an arousal. This Ach release triggers an activated awake state in corticothalamic system. Thus, an alteration affecting the IPN could result in arousal disorders.

The increased sensitivity of the mutated receptors (gain of function) cause increased activation of the frontal cortex trough corticothalamic connections. [46, 109]. In knockout mice of the nAChRs beta 2 subunit gene the organisation of sleep regulating microarousals was altered [111].

Based on the above mentioned data we can assume that, in ADNFLE, the hyper functioning mesopontine cholinergic pathway chronically overactivate the LDT and consequently the mediodorsal thalamic nucleus, by initiating arousals, activate different epileptic and non epileptic events.

NFLE have shown unusual features from the beginning of its discovery as “paroxysmal nocturnal dystonia” by the Lugaresi group [112] exhibiting variable motor fragments of arousal behaviour culminating eventually in night terror like frenetic storms of emotional behaviour. From the beginning the nocturnal events were described as “preceded” by arousal signs, independently of the degree of their repertoire. I propose here a change in the interpretation: to emphasize more that they are arousal responses with pathological motor, emotional and autonomic behavior, due to partial arousal in which the frontal dorsolateral cognitive faculties are deactivated by the presence of partial sleep (reflected by frontal convexity delta EEG).

### 3.4. Brain Mechanisms Explaining Epileptic and Non Epileptic Pathological Arousal Reactions during Sleep in NFLE and AP

The symptomatological similarities of the two continuums of paroxysmal nocturnal events in NFLE and arousal parasomnias (AP), the common relationship with the sleep process, and the genetic relationship between the two conditions led to look for a common pathological mechanism [97].

Nowadays our concepts about the nature of sleep are changing very much: the global nature of sleep has been challenged by data pointing to the existence of local/regional features of sleep (and wake) states. In some of the brain diseases elements of different vigilance states can occur simultaneously. For example in “REM behavior disorder” (connected often to Parkinson disease) motor actions occur during REM sleep. The recognition of these “mixed states” led to term “status dissociatus” [113]. APs are quite well fitting into this state dissociation concept being mixed conditions with Janus faced, partially awake and partially sleeping behavior. Experimental works showing the existence of sleeping cortical columns among others being in awake state contributed much to the local sleep concept [114]. Another approach has shown local changes of delta power after local utilisation of body functions during the previous day [115]. Alternating sleep of the two hemispheres discovered in dolphins and other aquatic mammals [116, 117] pushed thinking also toward the possibility of simultaneous mixed, awake- and sleep-like regions in the brain during certain sleep conditions.

In the last years two publications have provided congruent objective evidences for such dissociated conditions in APs patients. Bassetti et al. [118] reported a single Photon Emission Computed Tomography (SPECT) study in a man with AP. Their findings suggest a clear state dissociation: activation of thalamocingulate pathways and persisting deactivation of the frontodorsal region during a nocturnal episode.

Almost the same was demonstrated by Terzaghi et al. [119] in a patient under invasive presurgical evaluation for epilepsy who had a history of AP episodes and they were lucky to observe one event with implanted electrodes. During this episode in NREM sleep the frontal convexity showed the usual slow wave activity, but the cingulate gyrus emanated awake-like activity.

Similar mechanism can be offered as explanation to the night sleep events of NFLE and AP. The exaggerated arousals accompanied by confused behavior with autonomic signs and hypermotor automatisms are expressions of a different degree alarm-like behavior activated by the frontal cholinergic arousal system during the condition of depressed cognition in NREM sleep. This may happen either if the Ach receptors of the arousal system are mutated resulting in an overexcited arousal system in ADNFLE or, without epileptic disorder in AP, by a presently unknown mechanism.

Summing up the symptoms of idiopathic NFLE and its nonepileptic counterpart, the AP can be interpreted as pathologically exaggerated arousal reactions possibly related to mutation of the Ach nicotinic receptor subunit expressed at several levels of the ascending reticular arousal system, with persisting deactivation of the dorsolateral frontal cognitive functions due to NREM sleep.

## 4. Are the Major Frontal Lobe Epilepsies (Nocturnal Frontal Lobe Epilepsy and Absence Epilepsy) Disorders of Antagonistic Thalamofrontal Twin Sleep/Arousal Systems?

In the previous part of this paper two hypotheses have been elaborated:

- Absence epilepsy (AE) is an epileptic disorder of the corticothalamic system's burst-firing working mode, which under physiological circumstances produces NREM sleep EEG elements: spindling and delta activity.
- NFLE epilepsy is an epileptic facilitation of the arousal function during sleep due to a gain mutation in the cholinergic ascending reticular arousal system and has a nonepileptic counterpart: arousal parasomnia. Both disorders show a spectrum of paroxysmal night events from simple fragments of arousal behaviour to frenetic autonomic, motor, and emotional complex alarm behaviour, due to different degree of arousal with missing frontal dorsolateral cognitive functions during NREM sleep.

**Figure 3:**
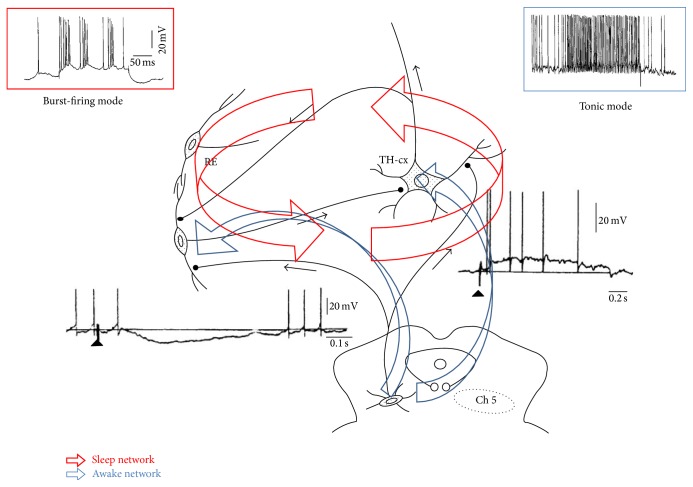
The thalamocortical system and its brainstem connections contain two antagonistic loops: one is responsible for the suppression-burst firing (red insert), and intrathalamic circuit connecting reticular inhibitory nuclei with thalamocortical relay neurons producing spindles and slow waves (sleep network); the other system is the ascending reticular system and thalamic connections (blue) conveying cholinergic arousal influences and inhibiting the reticular nuclei providing tonic thalamocortical neuronal discharge flow (blue upper insert), maintaining wake state. The right lower insert shows the excitatory postsynaptic effect of ascending cholinergic reticular neurons on the thalamocortical relay cell. The left lower insert shows the inhibitory postsynaptic effect of the cholinergic ascending arousal neurons on the thalamic reticular neurons (composed after Steriade [45], and Itier and Bertrand [46]).

Both AE and NFLE seem to be system epilepsies of the sleep-wake system. In other words these idiopathic major thalamofrontal epilepsies may represent epileptic distortions of the antagonistic twin (yin-yang type) sleep/arousal network (Figure 3).

This hypothesis fits into the endeavour to conceptualize epilepsies in physiologically meaningful networks.

## Highlights

1. “System epilepsy” is a new concept to define the relationship of certain epilepsies with physiological brain systems. Extended model of reflex-epilepsy is applied to associate NREM sleep system with AE and ascending cholinergic arousal system with NFLE.
2. Experimental research have proven that absence epilepsy with spike-wave pattern and the burst-firing mode of the corticothalamic system during NREM sleep share the same functional structures in the brain. Clinical research have shown that absences occur across the sleep-wakefulness continuum when the vigilance decreases, in transition states linked to shifts from wake state toward NREM sleep. Absences are inhibited by full awakeness and REM sleep. During the transition states those sensory stimuli which elicit slow wave response (reactive slow waves, ie CAP A1 phase) may activate spike-wave responses, while those eliciting traditional desynchronizational arousal responses do not.
3. Therefore, AE in terms of states and functions seems to be linked to initiation of NREM sleep.
4. ADNFLE, the genetic variation of NFLE, is underpinned by mutation of the nicotinic acetylcholine receptor a4 (CHRNA4) and b2 (CHRNB2) subunit on the 20q13 and 15q24 chromosome. This gain mutation renders the cholinergic ascending arousal system hypersensitive to microarousals during NREM sleep resulting in different degree of arousal linked seizure fragments and full seizures with behavioral manifestation from banal arousals to frenetic alarm reactions with hypermotor features. Semiology and occurrence pattern of seizures can be explained by increased propensity to arousal and dissociation between partially activated brain and sleeping (dysfunctional) dorsofrontal cortical system. The clinical symptoms of the genetic variation and of the more frequent sporadic cases do not differ.
5. Therefore, NFLE seems to be linked with the cholinergic arousal system under epileptic facilitation, with genetic or assumed genetic origin.
6. Arousal parasomnias show similar behavioral manifestations without epileptic nature. The two (epileptic and nonepileptic) disorders show strong familiar co- and cross-existence and probably share pathomechanism with the same dissociative characteristics.
7. Therefore, NFLE and the APs (as nonepileptic counterpart) both can be linked to the disorder of the cholinergic arousal system.
8. Based on the above described data and concepts we propose that both AE and NFLE are system epilepsies, and these idiopathic major thalamofrontal epilepsies may represent epileptic disorders of the antagonistic twin sleep/arousal network.
